# Sex differences in respiratory and circulatory cost during hypoxic walking: potential impact on oxygen saturation

**DOI:** 10.1038/s41598-019-44844-6

**Published:** 2019-07-02

**Authors:** Masahiro Horiuchi, Yoko Kirihara, Yoshiyuki Fukuoka, Herman Pontzer

**Affiliations:** 1 Division of Human Environmental Science, Mt. Fuji Research Institute, Kamiyoshida 5597-1, Fujiyoshida-city, Yamanashi 4030005 Japan; 2 Faculty of Health and Sports Science, Doshisha University, Tatara 1-3, Kyotanabe, Kyoto 6100394 Japan; 3 Department of Evolutionary Anthropology, Duke University, Biological Sciences Building Campus Box 90383, Durham, NC 27708-9976 USA

**Keywords:** Blood flow, Respiration

## Abstract

Energy expenditure (EE) during treadmill walking under normal conditions (normobaric normoxia, 21% O_2_) and moderate hypoxia (13% O_2_) was measured. Ten healthy young men and ten healthy young women walked on a level (0°) gradient a range of speeds (0.67–1.67 m s^−1^). During walking, there were no significant differences in reductions in arterial oxygen saturation (SpO_2_) between the sexes. The hypoxia-induced increase in EE, heart rate (HR [bpm]) and ventilation (${\dot{{\rm{V}}}}_{{\rm{E}}}$ [L min^−1^]) were calculated. Using a multivariate model that combined EE, ${\dot{{\rm{V}}}}_{{\rm{E}}}$, and HR to predict ΔSpO_2_ (hypoxia-induced reduction), a very strong fit model both for men (r^2^ = 0.900, *P* < 0.001) and for women was obtained (r^2^ = 0.957, *P* < 0.001). The contributions of EE, VE, and HR to ΔSpO_2_ were markedly different between men and women. ${\dot{{\rm{V}}}}_{{\rm{E}}}$ and EE had a stronger effect on ΔSpO_2_ in women (${\dot{{\rm{V}}}}_{{\rm{E}}}$: 4.1% in women vs. 1.7% in men; EE: 28.1% in women vs. 15.8% in men), while HR had a greater effect in men (82.5% in men and 67.9% in women). These findings suggested that high-altitude adaptation in response to hypoxemia has different underlying mechanisms between men and women. These results can help to explain how to adapt high-altitude for men and women, respectively.

## Introduction

Humans moved into high-altitude regions over the past 20,000 years. At high altitude, a reduction of alveolar PO_2_ limits pulmonary O_2_ diffusion capability, leading to a reduction in arterial O_2_ saturation (SpO_2_)^1,2^. Maintaining SpO_2_ at high-altitude may present an important challenge for populations living at altitude. Cardiovascular adaptation to altitude has been primarily studied at rest^3^, but comparatively little is known about metabolic responses during walking under hypoxia, particularly with regard to anthropometric and sex effects. Previous studies have suggested that the cost of breathing may differ between men and women. Women have smaller lungs and a decreased capacity for lung diffusion compared to age- and height-matched men^4–6^, as well as airways with smaller diameters when matched for lung volume^7,8^. These anatomical and functional sex differences may increase the work required for women to maintain a given rate of pulmonary ventilation (${\dot{{\rm{V}}}}_{{\rm{E}}}$)^9–14^. Consistent with this view, Dominelli and colleagues found that oxygen uptake of the respiratory muscle which can increase or decrease thorax (e.g., thoracic diaphragm, intercostales interni- and externi muscles), was significantly greater in women than in men at >55 L min^−1^ ${\dot{{\rm{V}}}}_{{\rm{E}}}$ during exercise^12^.

If breathing is more mechanically challenging for women, exercise-induced arterial hypoxemia may occur more readily in women than in men^15–20^. However, breathing mechanics and cost might not be the primary factors determining exercise-induced arterial hypoxemia. Circulatory dynamics could also affect oxygen saturation because oxygen saturation is the fraction of oxygen-saturated hemoglobin relative to the total hemoglobin in the blood. Thus, investigations of hypoxemia and its effects should consider both breathing and circulatory responses (e.g., heart rate; HR) on exercise-induced arterial hypoxemia.

Recently, to evaluate the energy cost of ventilation and circulation during hypoxic walking, an experimental model was developed and tested in healthy, young male lowlanders^21^. An important limitation of that study^21^ was that it included only men. It remains uncertain whether ventilation and circulation costs differ in women.

In this study, a hypoxemia model to investigate sex differences in ventilation and circulation costs and in SpO_2_ was employed. Men and women walked at a range of speeds under normobaric hypoxic conditions while measuring energy expenditure (EE), ${\dot{{\rm{V}}}}_{{\rm{E}}}$, and HR. Given the established sex differences in respiratory anatomy and work, it was hypothesized that women would experience greater arterial hypoxemia, and that the contribution rate of EE, ${\dot{{\rm{V}}}}_{{\rm{E}}}$, and HR in response to changes in SpO_2_ would be different between the sexes.

## Results

Table 1 shows the physical characteristics, respiratory function, and peak blood lactate concentration (La) at 5 min after walking. The absolute values of forced vital capacity (FVC) and FVC_1.0_ in men were significantly greater than in women. Predicted values (% predicted) adjusted by sex, age, and height were calculated based on the formula by Kubota *et al*. for Japanese populations^22^, and no significant differences in these variables between the sexes were observed. Resting La was similar across sexes in all oxygen conditions (1.2 ± 0.2 mmol L^−1^ in men vs. 1.2 ± 0.2 mmol L^−1^ in women under normoxia; 1.2 ± 0.2 mmol L^−1^ in men vs. 1.2 ± 0.3 mmol L^−1^ in women under hypoxia; all *P* > 0.05). There were no significant differences in peak La at 5 min after walking between the sexes within each oxygen condition.

**Table 1:** Comparison of the physical characteristics, respiratory functions, and peak blood lactate (La) concentrations between men and women.

	Men (n = 10)	Women (n = 10)	t (df = 18)	*P* value
Age (years)	22.6 ± 4.1	23.9 ± 5.8	−0.58	0.569
Height (m)	1.73 ± 0.06	1.58 ± 0.05*	5.78	<0.001
Body mass (kg)	66.2 ± 6.7	54.9 ± 5.4*	4.13	<0.001
BMI (kg m^2^)	22.1 ± 1.9	21.9 ± 2.2	0.20	0.847
BSA (m^2^)	1.789 ± 0.109	1.549 ± 0.083*	5.53	<0.001
FVC (L)	4.59 ± 0.46	3.20 ± 0.34*	7.58	<0.001
FVC (% predicted)	96.1 ± 9.9	98.4 ± 10.0	−0.52	0.608
FEV_1.0_ (L)	4.13 ± 0.39	2.87 ± 0.33*	7.82	<0.001
FEV_1.0_ (% predicted)	99.3 ± 10.3	99.1 ± 11.4	0.05	0.956
FEV_1.0_/FVC (%)	90.2 ± 2.6	89.5 ± 3.4	0.50	0.623
Peak La in N (mmol L^−1^)	1.7 ± 0.5	1.9 ± 0.7	−1.00	0.332
Peak La in H (mmol L^−1^)	3.9 ± 1.5	4.7 ± 1.3	−1.22	0.239

Values are mean ± standard deviation (SD). BMI: body mass index; BSA: body surface area; FVC: forced vital capacity; FEV_1.0_: forced expired volume in 1 s; La, blood lactate concentration, N, normoxia; H, hypoxia. Peak La was measured at 5 min after walking. *P < 0.05 between men and women.

**Figure 1:**
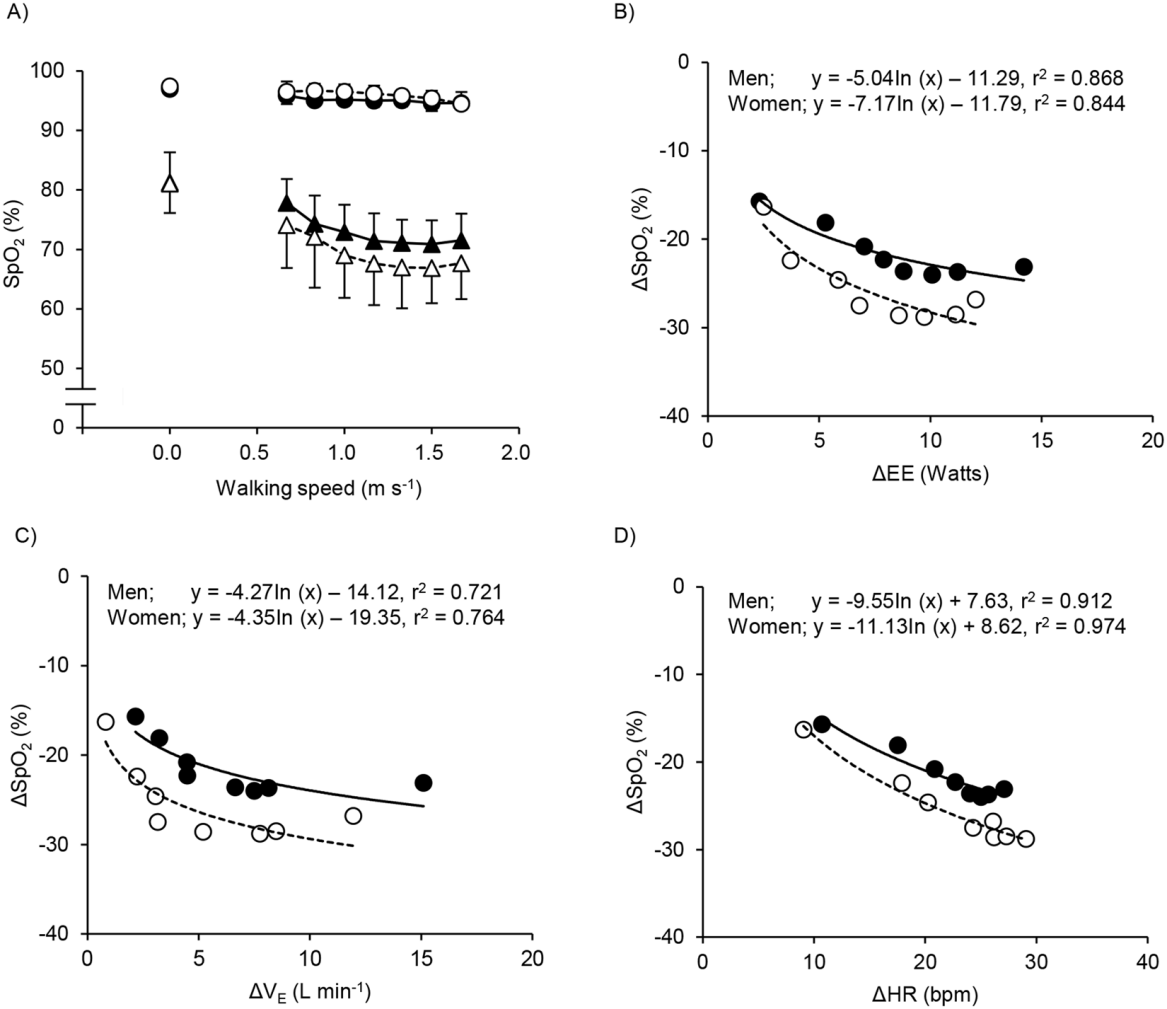
Changes in arterial oxygen saturation (SpO_2_; panel A), in ΔSpO_2_ versus in changes Δ energy expenditure (EE; panel B), versus changes in Δ pulmonary ventilation (V_E_; panel C), and versus in Δ heart rate (HR; panel D) between men and women at rest and each walking speed in normoxia and hypoxia are shown In the panel A, the number of 0.0 on the X axis indicate at rest, and values are mean ± standard deviation (SD). Closed symbols indicate men, and open symbols indicate women. Circles indicate normoxia, and triangles indicate hypoxia. In the panel B–D, logarithmic function curve are shown for between ΔSpO_2_ and ΔEE, between ΔSpO_2_ and ΔEE, and between ΔSpO_2_ and ΔHR. Closed symbols indicate men, and open symbols indicate women.

The time course changes in SpO_2_ in both sexes and oxygen conditions are shown (Fig. 1A). A significant main effect for time was observed in normoxia [F(7, 126) = 19.88, *P* < 0.001] and hypoxia [F(7, 126) = 55.08, *P* < 0.001], but there was no main effect for sex in normoxia [F(1, 18) = 3.96, *P* = 0.062] and hypoxia [F(1, 18) = 1.91, *P* = 0.184] and no interaction between sex and oxygen condition in normoxia [F(7, 126) = 1.65, *P* = 0.127] and hypoxia [F (7, 126) = 1.34, *P* = 0.236]. In normoxia, SpO_2_ decreased slightly ~3% from the resting baselines in both sexes. In contrast, SpO_2_ in hypoxia decreased greater compared to normoxia (~10% in men and ~14% in women) from the resting baselines. Results of bivariate analyses of ΔSpO_2_ (SpO_2 hypo_ − SpO_2 norm_) revealed significant correlations with ΔEE, Δ${\dot{{\rm{V}}}}_{{\rm{E}}}$, and ΔHR (second order polynomial regression) in men and women, respectively (Fig. 1B–D).

**Figure 2:**
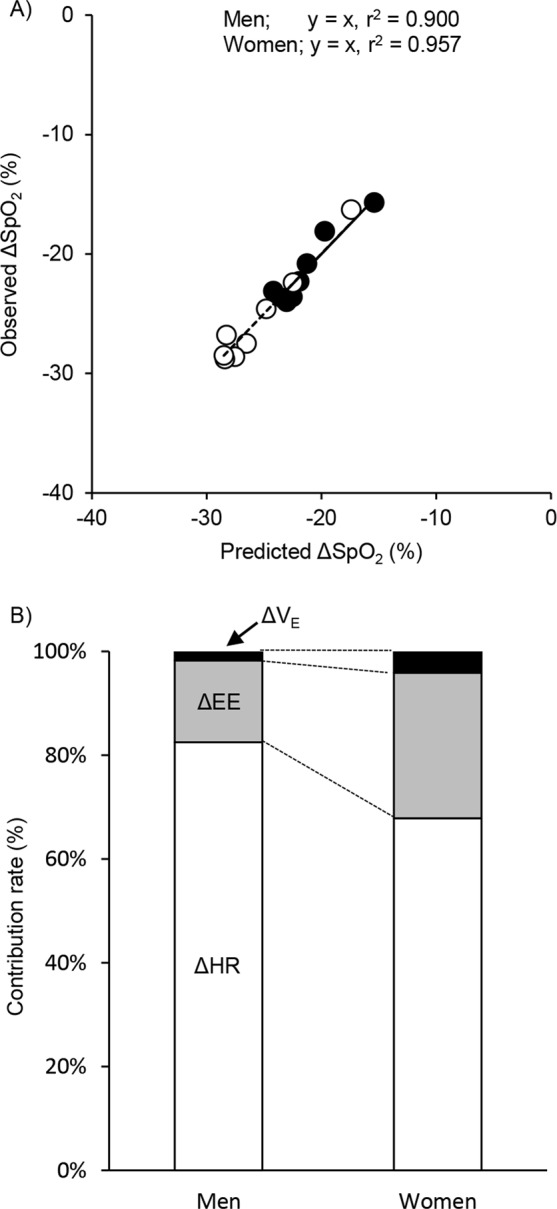
Observed ΔSpO_2_ plotted against that predicted ΔSpO_2_ from the least squares regression after logarithmic transformation for ΔEE, Δ${\dot{{\rm{V}}}}_{{\rm{E}}}$, and ΔHR: ΔSpO_2_ = −3.78ΔEE − 0.48ΔV_E_ − 13.45ΔHR for men and ΔSpO_2_ = −9.18ΔEE + 1.64ΔV_E_ − 14.21ΔHR for women; see text (upper panel). Line indicates y = x. Each symbol are the same as in Fig. 2. Contributions of EE (gray segments), ${\dot{{\rm{V}}}}_{{\rm{E}}}$ (black segments) and HR (white segments) to predict changes in SpO_2_ are shown (lower panel).

When log_10_ transformed values of ΔEE, Δ${\dot{{\rm{V}}}}_{{\rm{E}}}$, and ΔHR were included as independent variables in a multiple regression with ΔSpO_2_ as the dependent variable, ΔHR was found to be a significant factor in both men (t (8) = −6.54, *P* = 0.001) and women (t (8) = −4.04, *P* = 0.010). However, neither ΔEE nor Δ${\dot{{\rm{V}}}}_{{\rm{E}}}$ were significant factors for men or women (all *P* > 0.05). The model fit was very strong for men (ΔSpO_2_ = −3.78ΔEE − 0.48Δ${\dot{{\rm{V}}}}_{{\rm{E}}}$ − 13.45ΔHR, adj. r^2^ = 0.900, *P* < 0.001) and for women (ΔSpO_2_ = −9.18ΔEE + 1.64Δ${\dot{{\rm{V}}}}_{{\rm{E}}}$ − 14.21ΔHR, adj. r^2^ = 0.957, *P* < 0.001) (Fig. 2A).

Next, the contribution of ΔEE, Δ${\dot{{\rm{V}}}}_{{\rm{E}}}$, and ΔHR to the ΔSpO_2_ was calculated by using standard partial regression coefficients for men and women, separately. The relative contributions of these variables to ΔSpO_2_ was markedly different between men and women. In women, the effect of EE and ${\dot{{\rm{V}}}}_{{\rm{E}}}$ were greater (EE: 28.1% in women vs. 15.8% in men; ${\dot{{\rm{V}}}}_{{\rm{E}}}$: 4.1% in women vs. 1.7% in men). Conversely, in men the contribution of HR was greater (82.5% in men and 67.9% in women) (Fig. 2B).

**Figure 3:**
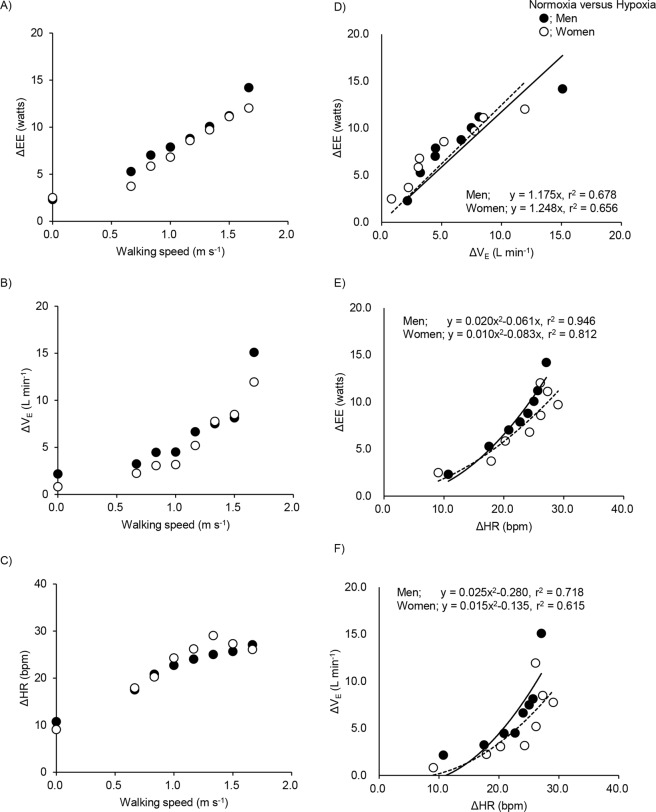
Mean values of differences in energy expenditure (EE; panel A), pulmonary ventilation (${\dot{{\rm{V}}}}_{{\rm{E}}}$; panel B), and heart rate (HR; panel C) between normoxia and hypoxia at rest and each walking speed between men and women. Changes in ΔEE versus in Δ${\dot{{\rm{V}}}}_{{\rm{E}}}$ (panel D), and changes in ΔEE versus in ΔHR (panel E), and changes in ΔHR and Δ${\dot{{\rm{V}}}}_{{\rm{E}}}$ (panel F) between men and women. Ordinary least squares linear regression equation, with an intercept = 0 are shown for between ΔEE and Δ${\dot{{\rm{V}}}}_{{\rm{E}}}$. Second- order polynomial regression equation, with an intercept = 0 are shown for between ΔEE and ΔHR, and between Δ${\dot{{\rm{V}}}}_{{\rm{E}}}$ and ΔHR. Each symbol are the same as in Fig. 1. Solid lines indicate for men and dashed lines indicate for women. The number of 0.0 on the X axis indicate at rest.

The mean values (±SD) of EE, EE, ${\dot{{\rm{V}}}}_{{\rm{E}}}$, and HR at rest and each walking speed, and changes in these variables with hypoxia are shown in Supplemental Table 1. ΔEE and ΔV_E_ in both men and women increased with speed; in particular, those in men were higher than in women at the fastest walking speed (Fig. 3A,B). Moreover, significant sex differences in breathing frequency and tidal volume were observed (Supplemental Table 2). ΔHR increased initially with walking speed before plateauing at higher walking speeds in both men and women (Fig. 3C). Significant correlations between ΔEE and Δ${\dot{{\rm{V}}}}_{{\rm{E}}}$ (linear regression analysis), and between ΔEE and ΔHR (second-order polynomial regression) were obtained with bivariate analyses (Fig. 3D,E). With the intercept set at 0, EE increased with the ventilatory effort as ΔEE = 1.175 ± 0.098Δ${\dot{{\rm{V}}}}_{{\rm{E}}}$ (r^2^ = 0.678, *P* < 0.05) in men, and ΔEE = 1.248 ± 0.111Δ${\dot{{\rm{V}}}}_{{\rm{E}}}$ (r^2^ = 0.656, *P* < 0.05) in women, which is approximately 1 Watt for every 1 L min^−1^ increment in ${\dot{{\rm{V}}}}_{{\rm{E}}}$ for both men and women (values are coefficient ± standard error). EE increased as a function of HR as ΔEE = 0.020 ± 0.004ΔHR^2^ − 0.061 ± 0.101ΔHR (r^2^ = 0.946, *P* < 0.001) in men and ΔEE = 0.010 ± 0.006ΔHR^2^ − 0.083 ± 0.159ΔHR (r^2^ = 0.812, *P* < 0.001) in women, which is approximately 1 Watt for every 8.8 beats per minute increased in HR for men and for every 15 beats per minute for women.

**Figure 4:**
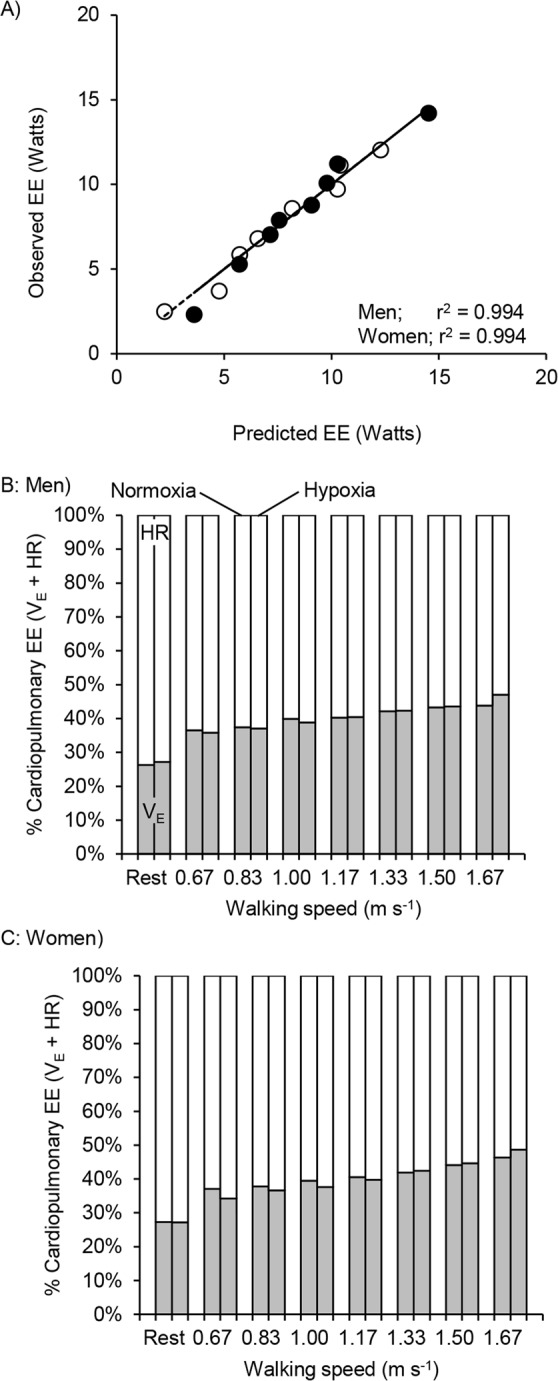
Observed ΔEE plotted against that predicted ΔEE from the least squares regression with ${\dot{{\rm{V}}}}_{{\rm{E}}}$ and HR: ΔEE = 0.564Δ${\dot{{\rm{V}}}}_{{\rm{E}}}$ + 0.222ΔHR for men and ΔEE = 0.613Δ${\dot{{\rm{V}}}}_{{\rm{E}}}$ + 0.190ΔHR for women; see text (panel A). Line indicates y = x. Each symbol and line are the same as in Fig. 2. Contributions of ${\dot{{\rm{V}}}}_{{\rm{E}}}$ (gray segments) and HR (white segments) to cardiopulmonary energy expenditure at rest and each walking speed under normoxia and hypoxia conditions between men (panel B) and women (panel C).

As Δ${\dot{{\rm{V}}}}_{{\rm{E}}}$ and ΔHR were correlated by second order polynomial regression analysis (r^2^ = 0.718 in men; r^2^ = 0.615 in women, Fig. 3F), it is difficult to determine their independent effects on EE. When Δ${\dot{{\rm{V}}}}_{{\rm{E}}}$ and ΔHR were included as independent variables in a multiple regression for men and women, separately, both Δ${\dot{{\rm{V}}}}_{{\rm{E}}}$ (t (8) = 6.19, *P* < 0.001 in men and t (8) = 7.16, *P* < 0.001 in women) and ΔHR (t (8) = 7.22, *P* < 0.001 in men and t (8) = 8.11, *P* < 0.001 in women) achieved statistical significance. The model fitted in very strongly (ΔEE = 0.564Δ${\dot{{\rm{V}}}}_{{\rm{E}}}$ + 0.222ΔHR, adj. r^2^ = 0.994, *P* < 0.001 in men, and ΔEE = 0.613Δ${\dot{{\rm{V}}}}_{{\rm{E}}}$ + 0.190ΔHR, adj. r^2^ = 0.994, *P* < 0.001 in women, respectively). When both HR and ${\dot{{\rm{V}}}}_{\dot{{\rm{E}}}}$ are included as independent variables in multivariate regression predicting ΔEE their individual effect sizes are each smaller than in separate bivariate analyses. Ventilatory costs were approximately 1 Watt for every 1.8 L min^−1^ increment (men) and 1.6 L min^−1^ increment (women) in ${\dot{{\rm{V}}}}_{{\rm{E}}}$; circulatory costs were approximately 1 Watt for every 4.5 beats per minute for men and 5.3 beats per minute for women. Figure 4A shows the model fit for all data.

We estimated the energy expenditure on cardiopulmonary work during normoxic and moderate hypoxia walking trials by imputing ${\dot{{\rm{V}}}}_{{\rm{E}}}$ and HR values for each condition into sex-specific models combining ventilatory and circulatory costs (ΔEE = 0.564Δ${\dot{{\rm{V}}}}_{{\rm{E}}}$ + 0.222ΔHR for men; ΔEE = 0.613Δ${\dot{{\rm{V}}}}_{{\rm{E}}}$ + 0.190ΔHR for women). Estimated cardiopulmonary energy cost at rest was roughly one-third of total EE (Watts) across the sexes and conditions. As walking speed increased, moderate hypoxia accentuated the increases in EE. Moreover, as walking energy expenditure increased with speed the portion attributed to cardiopulmonary work decreased across the sexes and conditions. The differences of EE or %EE between men and women were roughly 5% (EE) or 2% (%EE) at rest and during walking in both normoxia and hypoxia (Table 2).

**Table 2:** Mean values of EE (Watts), the percentage EE to total cardiopulmonary work (% EE), the percentage of net cardiopulmonary EE (% walk net), the difference between normoxia and hypoxia (Norm vs. Hypo) in men and women, and the difference between in men and women under normoxia and hypoxia at rest and each walking speed under all conditions.

Watts, W	Men (n = 10)		Women (n = 10)		Hypo - Norm		Women - Men	
	Normoxia	Hypoxia	Normoxia	Hypoxia	Men	Women	Norm	Hypo
0.67 m s^−1^	31.1	36.8	28.1	32.0	5.7	3.9	−3.0	−4.8
0.83 m s^−1^	33.2	40.3	30.2	35.8	7.1	5.6	−8.0	−4.5
1.00 m s^−1^	36.7	44.2	32.4	38.9	7.5	6.5	−4.3	−5.3
1.17 m s^−1^	39.2	48.2	35.5	43.3	9.0	7.8	−3.7	−4.9
1.33 m s^−1^	43.2	53.0	38.7	48.7	9.8	10.0	−4.5	−4.3
1.50 m s^−1^	47.5	57.8	44.1	53.9	10.3	9.8	−3.4	−3.9
1.67 m s^−1^	52.5	67.0	50.3	62.3	14.5	12.0	−1.8	−4.7
**% EE, %**								
0.67 m s^−1^	12.0	13.9	14.0	15.7	1.9	1.7	2.0	1.8
0.83 m s^−1^	11.1	13.2	13.8	15.9	2.1	2.1	2.7	2.7
1.00 m s^−1^	11.0	13.0	13.3	15.5	2.0	2.2	2.3	2.5
1.17 m s^−1^	10.6	12.8	12.8	15.1	2.2	2.3	1.8	2.3
1.33 m s^−1^	10.4	12.4	12.1	14.8	2.0	2.7	1.7	2.4
1.50 m s^−1^	9.9	11.8	11.8	14.0	2.0	2.2	1.9	2.2
1.67 m s^−1^	9.6	12.0	11.4	13.7	2.4	2.3	1.8	1.7
**% walk net, %**								
0.67 m s^−1^	4.0	5.3	6.0	7.7	1.3	1.7	2.0	2.4
0.83 m s^−1^	4.2	6.0	6.8	9.4	1.8	2.6	2.6	3.4
1.00 m s^−1^	5.2	6.8	7.2	9.8	1.6	2.6	2.0	3.0
1.17 m s^−1^	5.4	7.4	7.5	10.2	2.0	2.7	2.1	2.8
1.33 m s^−1^	5.9	7.7	7.5	10.7	1.8	3.2	1.6	3.0
1.50 m s^−1^	6.1	7.7	8.0	10.4	1.6	2.4	1.9	2.7
1.67 m s^−1^	6.3	8.6	8.2	10.7	2.3	2.5	1.9	2.1

“Hypo – Norm” and “Women – Men” indicate the values in hypoxia minus those in normoxia, and the values in women minus those in men, respectively.

To calculate the contribution of cardiopulmonary expenditure to net walking cost, we subtracted EE and estimated cardiopulmonary cost at rest from values measured during walking. Net cardiopulmonary cost accounted for a slightly greater proportion of net EE in women in both normoxic (~2%) and hypoxic (~3%) conditions and. However, in both men and women the percentage of net EE attributable to cardiopulmonary expenditure increased by ~3% as walking speed increased 0.67 m s^−1^ to 1.67 m s^−1^ (Table 2).

Finally, using the multivariate model for cardiopulmonary costs, we calculated the relative contribution of ventilation and circulation to total cardiopulmonary expenditure. The estimated energy cost of circulation (HR) exceeded that of respiration (${\dot{{\rm{V}}}}_{{\rm{E}}}$) in both normoxia and hypoxia. HR accounted for more than 70% of cardiopulmonary work at rest in both sexes and oxygen conditions. As walking speed increased, the proportion of cardiopulmonary work attributable to HR decreased in all conditions. These tendencies are similar between men and women (Fig. 4B,C).

## Discussion

Although significant reductions in SpO_2_ during walking were observed both in normoxia and hypoxia in the present study, no sex differences in SpO_2_ changes were found. Reduction in SpO_2_ during exercise (i.e., exercise-induced arterial hypoxemia; EIAH) has been attributed to the mechanical ventilatory limits of the respiratory system^18,23^. This result run counter to expectations from womens’ smaller respiratory anatomy and greater mechanical ventilatory constraints^4^ which would predict more severe or frequent EIAH women than in men^15–20^. Given that other studies have also shown no differences in EIAH between the sexes^24,25^, the role of mechanical constraints in EIAH should be reassessed.

One possible explanation to account for the variability in the detection of EIAH across studies is the different study settings (e.g., exercise mode and intensity). In these previous studies, subjects performed leg cycling exercise until exhaustion in normoxia^15,17–20^ or hypoxia^25^, while the present study settings were performed with relatively lower exercise intensity (i.e., treadmill walking) in hypoxia. In addition, subjects’ training status should also be considered, e.g., trained women^20^, untrained men *vs*. trained women^25^, trained *vs*. untrained women^15,17,19^, and untrained men *vs*. women in the present study. Given the very different experimental designs, these controversial findings may not be surprising.

In women, the contribution of ventilatory work and EE on SpO_2_ was approximately twice that found in men based on modified analysis model^21^. It has been reported that oxygen cost of breathing for a given ${\dot{{\rm{V}}}}_{{\rm{E}}}$ was greater in women compared to men^9–14^, probably, due to smaller lung size^4–6^ and airway diameters^7,8^. Additionally, a recent study demonstrated that electromyography of the extra-diaphragmatic inspiratory muscles (i.e., sternocleidomastoid, and scalene muscles) was significantly greater in women than in men at all submaximal time points and at maximal exercise during incremental leg cycling despite lower ${\dot{{\rm{V}}}}_{{\rm{E}}}$ in women compared to men^26^. The effects of ${\dot{{\rm{V}}}}_{{\rm{E}}}$, breathing frequency and tidal volume should also be considered. In the present study, women have greater respiration frequency compared to men, resulting in greater tidal volume in men than women (Supplemental Table 2). A previous study demonstrated that slow deep breathing increased SpO_2_ levels probably, induced by improvement in ventilatory efficiency^27^. This has been suggested to be caused by a reduction in dead space ventilation and an increase in alveolar ventilation^28^. Together, these results support the idea that ${\dot{{\rm{V}}}}_{{\rm{E}}}$ plays a larger role in SpO_2_ changes for women.

It should be noted that experimental design of the present study controlled for the effects of menstrual cycle on ${\dot{{\rm{V}}}}_{{\rm{E}}}$. Estrogen and progesterone are both known to have a stimulatory effect on ${\dot{{\rm{V}}}}_{{\rm{E}}}$ ^29,30^; resting ${\dot{{\rm{V}}}}_{{\rm{E}}}$ is higher or the same in luteal phase of the cycle when progesterone concentrations show highest than follicular phase^31,32^ and resting ${\dot{{\rm{V}}}}_{{\rm{E}}}$ responsiveness to hypoxia is sensitive to progesterone levels^32–34^. To minimize menstrual cycle effects, all women included in the present study were measured during the early follicular phase of their menstrual cycle.

It was also found that the contribution of EE to SpO_2_ changes in women is twice that of men. It has been reported that at ~55 L min^−1^ ${\dot{{\rm{V}}}}_{{\rm{E}}}$, oxygen uptake ($\dot{{\rm{V}}}$O_2_) of the respiratory muscle was significantly greater in women than in men^12^. Since experimental protocol of the present study only reached ~55 L min^−1^ ${\dot{{\rm{V}}}}_{{\rm{E}}}$ at the fastest gait speed in men, the values of ${\dot{{\rm{V}}}}_{{\rm{E}}}$ in the present study may be too low to detect greater oxygen uptake of the respiratory muscle in women. The previous study was conducted using voluntary hyperpnoea in normoxia^9–14^, by contrast, at faster gait speed during hypoxic walking, greater breathing frequencies and lower tidal volume might induce greater respiratory ${\dot{{\rm{V}}}{\rm{O}}}_{2}$ in women compared to men.

In the present study, we found that men and women had similar energy costs for ${\dot{{\rm{V}}}}_{{\rm{E}}}$ (1 Watt for every 1.2 L min^−1^ increment in men and women) but, different costs between the sexes for HR (1 Watt for every 9 beats per minute increment for men and 15 beats per minute for women). In our previous study in men^21^, we found greater ventilatory costs (1 Watt per 2.3 L min^−1^) and lower circulatory costs (1 Watt per 3 bpm). This discrepancy may simply reflect the limits of precision in our multivariate approach. Another possible explanation to account for these differences may be the different study settings [FiO_2_ = 15 and 11%O_2_ in the previous study^21^ vs. 13%O_2_ in the present study] and subject characteristics. In the previous study, the subjects were high-fit athletes^21^, while the subjects in the present study were sedentary and not athletes.

### Methodological considerations

There are several limitations in the present study. First, peak aerobic capacity was not directly evaluated, as it is often done. Thus, there is a possibility that relative exercise intensity may be different between the sexes and/or within subjects. To minimize this effect, sex-specific models were used, and resulted in similar changes in EE, ${\dot{{\rm{V}}}}_{{\rm{E}}}$ and HR (see Fig. 4A–C). Second, anatomical differences such as air way and lung size were not evaluated. Instead, anatomical interpretations of the present results are based on previous studies^4–8^ and should be treated with caution. Similarly, although a reduction in SpO_2_ has been thought to be related pulmonary O_2_ diffusion capacity^1,2^, a previous study found greater inter-individual differences in the alveolar-capillary membrane diffusing capacity and the pulmonary capillary blood volume, which are subcomponents of total lung diffusion capacity with an inspiration of different concentration of O_2_ (20-40-60% O_2_)^35^. Similar results (i.e., greater inter-indvidual differences in lung diffusion capacity) were found under hypobaric hypoxia at rest^36^ and during sub-maximal exercise^37^. In light of these previous studies, it is evident that lung diffusion capacity (which may affect SpO_2_) causes inter-individual differences under different barometric pressure condition and/or fraction of inspired oxygen. Including these measures in future work will advance our understanding of the sex differences in respiration and circulation costs as well as EIAH during hypoxic walking.

In conclusion, using a multivariate model that used EE, ${\dot{{\rm{V}}}}_{{\rm{E}}}$, and HR to predict ΔSpO_2_ (hypoxia-induced reduction), a very strong fit for both men (r^2^ = 0.900, *P* < 0.001) and women (r^2^ = 0.957, *P* < 0.001) was obtained. The relative contributions of EE, ${\dot{{\rm{V}}}}_{{\rm{E}}}$ and HR to ΔSpO_2_ were markedly different between the sexes. Specifically, the contribution of EE and ${\dot{{\rm{V}}}}_{{\rm{E}}}$ in women was about two-fold compared to men. Conversely, that of HR in men was greater than in women. These findings suggested that high-altitude adaptation in response to hypoxemia has different underlying mechanisms between men and women.

## Methods

### Participants

The participants were 10 young men and 10 young women, and their physical characteristics are shown in Table 1. All of the participants did not engage in regular exercise. They were free from any known cardiovascular diseases and had not taken any medications that affect cardiovascular responses. Participants were requested to abstain from caffeinated beverages and alcohol on the day before testing and intense physical activity two days before testing. All women were studied during the early follicular phase (days 1–5) of the menstrual cycle for both protocol (i.e., normoxia and hypoxia) where day 0 is the start of menstruation^38^. Women subjects, who had irregular menstrual cycles or were taking birth control medication, were excluded from the study. After a detailed description and explanation of all study procedures, possible risks and benefits of participation, each subject signed an informed consent form.

All procedures in the present study were approved by the ethical committee of Mount Fuji Research (No: ECMFRI-03-2014) in Japan and were performed in accordance with the guidelines of the Declaration of Helsinki.

### Study procedures

A motor-driven treadmill (Aero Walker 2200, Combi Wellness Co, Ltd., Tokyo, Japan) was used to conduct all experiments. Under all experimental conditions, participants walked on the same treadmill; they were free to choose their step frequency at each speed. All participants wore lightweight training shoes, socks, shirts, and underwear. All participants underwent a familiarization session to allow them to become accustomed to the treadmill walking while wearing a gas collection mask for at least three times at several walking speeds. A fraction of inspiratory oxygen (FiO_2_) was set at normobaric hypoxia (FiO_2_: 21%; room air at a 1,065 m altitude) and moderate hypoxia (FiO_2_: 13%; equivalent to a simulated altitude of 3,200 m, around at which the hemoglobin dissociation curve begins to decrease abruptly). The hypoxic gas was continuously blended using a hypoxic gas generator system (Everest summit II, Will Co. Ltd., Tokyo, Japan) and delivered from a 200 litre Douglas bag. Each trial (i.e., normoxia or hypoxia) was performed on different days in random order, and a single blind method was used. First, each participant sat in a chair for 10 min and then stood for 5 min on the treadmill for a baseline standing measurement. Then, the participants started to walk on the treadmill. Following preliminary tests in our laboratory and the modified protocol in the previous studies^21,36^, seven walking speeds used in ascending order (0.67, 0.83, 1.00, 1.17, 1.33, 1.50, and 1.67 m s^−1^). Each speed was maintained for four minutes.

### Measurement variables

Respiratory data were measured by a breath-by-breath gas analyzer (AE-310S, Minato Ltd., Osaka, Japan). Inspired and expired gas volumes were measured using a hot-wire respiratory flow system. Flow signals were electrically integrated for the duration of each breath to calculate minute ventilation. The expired fractions of O_2_ and CO_2_ were analyzed using a zirconium solid electrolyte oxygen analyzer and an infrared CO_2_ analyzer, respectively. The standard, known gases (O_2_ 15.23%, CO_2_ 4.999%, and N_2_ balance) and room air were used to calibrate the gas analyzer before each test. HR was measured with a commercial HR monitor (POLAR RC800X, POLAR Electro, Tokyo, Japan) throughout this study. SpO_2_ was continuously monitored by the pulse oximeter on the left middle finger, and it recorded every 1 min throughout the study (TM-2564G, A&D, Tokyo, Japan). Fingertip blood samples (0.3 μL) were collected to determine the La at rest and after 5 min of walking. La was measured using a lactate analyzer (Lactate Pro 2, Arkray, Kyoto, Japan). The resting respiratory function was assessed with a spirometer (CHESTGRAPH HI-105T, Chest, Tokyo, Japan). The measurement was performed when the subjects were in a sitting position. Before the test, they practiced at least three times and were familiar with a spirometer (e.g., maximal inspiration and expiration). We measured FVC and forced expiratory volume in one second (FEV_1.0_). Predicted FEV_1.0_, predicted FVC, and the ratio of these variables (FEV_1.0_/FVC) were calculated using the equation developed by the Japanese Respiratory Society^22^.

### Data analysis

EE at rest and each walking speed was calculated using $\dot{{\rm{V}}}$O_2_ and carbon dioxide output ($\dot{{\rm{V}}}$CO_2_) with the following equation:

1 $${\rm{EE}}\,({\rm{Watts}})=[(3.869\times {\dot{{\rm{V}}}{\rm{O}}}_{2}/1000)+(1.195\times {\dot{{\rm{V}}}\mathrm{CO}}_{2}/1000)]\times 4.186/60\times 1000.$$

Gas exchange variables, SpO_2_, and HR were averaged for the last 2 min of standing prior to walking. During walking, mean values of final 1 min at each walking speed of physiological data were also obtained^21,39,40^.

Respiratory and circulatory cost was evaluated using the same method as in a recent study^21^. Briefly, total walking energy expenditure at each speed (including rest) during normoxic trials (EE_norm_) were subtracted from the values during hypoxic trials at the same walking speed (EE_hyp_) to calculate a ΔEE value. We used a similar approach to calculate hypoxia-induced changes in ${\dot{{\rm{V}}}}_{{\rm{E}}}$ (Δ${\dot{{\rm{V}}}}_{{\rm{E}}}$ = ${\dot{{\rm{V}}}}_{{\rm{E}}{\rm{hyp}}}$ − ${\dot{{\rm{V}}}}_{{\rm{E}}{\rm{norm}}}$) and HR (ΔHR = HR_hyp_ − HR_norm_). These calculations were conducted separately for men and women. Then, ordinary least squares linear regression was used to compare ΔEE to Δ${\dot{{\rm{V}}}}_{{\rm{E}}}$ and to ΔHR separately (note: a second order polynomial regression equation was used with ΔHR). Next, to assess the independent, additive effects of ${\dot{{\rm{V}}}}_{{\rm{E}}}$ and HR on EE multiple regression was used (Cardiopulmonary Cost: ΔEE ~Δ${\dot{{\rm{V}}}}_{{\rm{E}}}$ + ΔHR). In these multiple regression analyses the intercept was set to 0 under the assumption that ΔEE was attributable to circulatory and ventilatory expenditure.

Similarly, changes in SpO_2_ with hypoxia (SpO_2 hypo_ − SpO_2 norm_) were calculated in men and women separately. Then, multiple regression analysis was used to test for the independent, additive effects of EE, ${\dot{{\rm{V}}}}_{{\rm{E}}}$, and HR on SpO_2_ after logarithmic transformation. This design yielded n = 8 comparisons (1 rest + 7 walking speeds for one hypoxic condition) for men and women, respectively.

Body mass index was calculated by body mass (kg) divided by the square values of height (m^2^). Body surface area (BSA) was also calculated with the following equation:

$${\rm{BSA}}={\rm{Height}}\,{({\rm{cm}})}^{0.725}\times {\rm{Body}}\,{\rm{mass}}\,{({\rm{kg}})}^{0.425}\times 0.007184$$

### Statistics

All data are expressed as mean ± standard deviation (SD). An unpaired t-test was performed in order to compare the physical characteristics, respiratory function, and La between men and women. To compare EE, ${\dot{{\rm{V}}}}_{{\rm{E}}}$, HR, SpO_2_, TV, and RR in normoxia and hypoxia, a two-way repeated ANOVA (sex × walking speed) was performed. Ventilatory or circulatory costs was calculated using a bivariate regression model as described. All statistical analyses were performed using commercial statistical software (Sigma Stat version 3.5, Chicago, IL, USA). The statistical significance was set as a *P* value of < 0.05^41^.

## Supplementary information

Supplemental Table 1 and 2
