# Supplementary material for: Sex differences in respiratory and circulatory cost during hypoxic walking: potential impact on oxygen saturation
Source: Sci Rep. 2019 Jul 2;9:9550. doi: 10.1038/s41598-019-44844-6 (PMC6606641; doi:10.1038/s41598-019-44844-6)
Supplement: Supplementary file 1 — Supplemental Table 1 and 2 [file 41598_2019_44844_MOESM1_ESM.pdf]

TITLE: Sex differences in respiratory and circulatory cost during hypoxic walking: potential impact on oxygen saturation

Masahiro Horiuchi[1], Yoko Kirihara[1], Yoshiyuki Fukuoka[2], Herman Pontzer[3]

1. Division of Human Environmental Science, Mt. Fuji Research Institute, Kamiyoshida 5597-, Fujiyoshida-city, Yamanashi, 4030005, Japan.

2. Faculty of Health and Sports Science, Doshisha University, Tatara 1-3, Kyotanabe, Kyoto, 6100394, Japan.

3. Department of Evolutionary Anthropology, Duke University, Biological Sciences Building Campus Box 90383 Durham, NC 27708-9976, USA

Corresponding author: Masahiro Horiuchi, Ph.D.

Kamiyoshida 5597-1, Fujiyoshida city, Yamanashi, Japan, 4030005

TEL; +81-555-72-6207, FAX; +81-555-72-6215

E-mail; mhoriuchi@mfri.pref.yamanashi.jp

Supplemental Table 1. Energy expenditure (EE), pulmonary ventilation ($\dot{V}_E$), and heart rate (HR) at rest and each gait speed under normoxia and hypoxia in men and women. Changes in these variables (Δ) under moderate hypoxia versus normoxia are shown.

	Men			Women		
	Normoxia	Hypoxia		Normoxia	Hypoxia	
EE, Watts			ΔEE			ΔEE
Rest	111 ± 14	114 ± 14	3	89 ± 15*	91 ± 13†	2
0.67m s^{-1}	259 ± 34	264 ± 40	5	200 ± 27*	204 ± 24†	4
0.83 m s^{-1}	299 ± 47	306 ± 50	7	219 ± 32*	224 ± 23†	5
1.00 m s^{-1}	332 ± 51	341 ± 52	9	244 ± 35*	251 ± 24†	7
1.17 m s^{-1}	368 ± 63	377 ± 62	9	278 ± 37*	287 ± 29†	9
1.33 m s^{-1}	417 ± 67	427 ± 65	10	320 ± 41*	329 ± 32†	9
1.50 m s^{-1}	478 ± 74	489 ± 67	11	375 ± 56*	386 ± 50†	11
1.67 m s^{-1}	545 ± 74	559 ± 67	14	443 ± 61*	455 ± 55†	12
$\dot{V}_E$, L min^{-1}			$\Delta\dot{V}_E$			$\Delta\dot{V}_E$
Rest	11.7 ± 2.9	13.9 ± 4.3	2.2	9.5 ± 1.5	10.4 ± 1.6†	0.9
0.67m s^{-1}	20.1 ± 3.6	23.3 ± 3.9	3.2	17.0 ± 2.1	19.2 ± 2.5	2.2
0.83 m s^{-1}	22.0 ± 7.4	26.4 ± 3.8	4.4	18.6 ± 3.1	21.7 ± 2.7	3.1
1.00 m s^{-1}	25.9 ± 6.0	30.4 ± 4.6	4.5	20.9 ± 3.3	24.1 ± 2.5	3.2
1.17 m s^{-1}	28.0 ± 5.7	34.6 ± 6.2	6.6	23.5 ± 3.4	28.7 ± 3.9	5.2
1.33 m s^{-1}	32.2 ± 6.1	39.7 ± 6.3	7.5	26.4 ± 3.8	34.2 ± 4.4	7.8
1.50 m s^{-1}	36.5 ± 6.6	44.6 ± 7.4	8.1	31.8 ± 4.9	40.3 ± 5.9	85
1.67 m s^{-1}	40.7 ± 7.2	55.8 ± 9.9	15.1	38.1 ± 5.1	50.0 ± 6.9	11.9
HR, bpm			ΔHR			ΔHR
Rest	84 ± 8	94 ± 9	10	82 ± 10	91 ± 10	9
0.67m s^{-1}	89 ± 7	107 ± 10	18	93 ± 10	111 ± 11	18
0.83 m s^{-1}	94 ± 7	115 ± 12	21	99 ± 12	119 ± 14	20
1.00 m s^{-1}	99 ± 6	122 ± 14	23	103 ± 13	128 ± 12	25
1.17 m s^{-1}	106 ± 6	130 ± 16	24	111 ± 13	137 ± 11	26
1.33 m s^{-1}	113 ± 7	138 ± 15	25	118 ± 15	147 ± 9	29
1.50 m s^{-1}	122 ± 8	147 ± 16	25	130 ± 17	157 ± 7	27
1.67 m s^{-1}	133 ± 13	160 ± 15	27	142 ± 17	168 ± 6	26

Results of a two-way ANOVA

	Normoxia	Hypoxia
EE	Sex: $F_{(1,18)} = 16.71$, $P < 0.001$	Sex: $F_{(1,18)} = 19.57$, $P < 0.001$
	Speed: $F_{(7,126)} = 532.67$, $P < 0.001$	Speed: $F_{(7,126)} = 700.52$, $P < 0.001$
	Interaction: $F_{(7,126)} = 6.64$, $P < 0.001$	Interaction: $F_{(7,126)} = 8.40$, $P < 0.001$
$\dot{V}_E$	Sex: $F_{(1,18)} = 4.06$, $P = 0.059$	Sex: $F_{(1,18)} = 6.59$, $P = 0.019$
	Speed: $F_{(7,126)} = 297.86$, $P < 0.001$	Speed: $F_{(7,126)} = 352.39$, $P < 0.001$
	Interaction: $F_{(7,126)} = 1.42$, $P = 0.202$	Interaction: $F_{(7,126)} = 0.524$, $P = 0.815$
HR	Sex: $F_{(1,18)} = 1.08$, $P = 0.313$	Sex: $F_{(1,18)} = 1.21$, $P = 0.286$
	Speed: $F_{(7,126)} = 271.72$, $P < 0.001$	Speed: $F_{(7,126)} = 578.43$, $P < 0.001$
	Interaction: $F_{(7,126)} = 2.29$, $P = 0.032$	Interaction: $F_{(7,126)} = 5.00$, $P < 0.001$

Values are mean ± standard deviation (SD). bpm; beats per minute. A two way repeated measures analysis of variance (ANOVA) was performed for a comparison between men and women within normoxia or hypoxia, respectively.* $P < 0.05$ between men and women in normoxia. † $P < 0.05$ between men and women in hypoxia.

Supplemental Table 2. Breathing frequency (*Bf*) and tidal volume (TV) at rest and each gait speed under normoxia and hypoxia in men and women.

	Normoxia		Hypoxia	
	Men	Women	Men	Women
Bf (frequency)				
Rest	11.2 ± 3.4	8.7 ± 1.9	12.1 ± 3.6	10.2 ± 3.0
0.67m s^{-1}	19.2 ± 4.8	20.2 ± 2.3	18.6 ± 3.7	18.4 ± 3.4
0.83 m s^{-1}	19.1 ± 6.8	21.4 ± 3.9	19.3 ± 4.6	21.0 ± 3.1
1.00 m s^{-1}	19.6 ± 4.6	23.1 ± 4.2	23.5 ± 5.7	24.7 ± 4.6
1.17 m s^{-1}	20.1 ± 5.4	25.6 ± 4.1*	24.9 ± 4.3	28.5 ± 4.6
1.33 m s^{-1}	22.7 ± 5.5	27.6 ± 5.1	26.3 ± 3.8	32.8 ± 4.8†
1.50 m s^{-1}	23.7 ± 6.1	31.9 ± 5.5*	30.4 ± 4.6	35.4 ± 4.5†
1.67 m s^{-1}	26.8 ± 4.7	32.4 ± 4.7*	33.5 ± 5.0	38.1 ± 4.2†
TV (L min^{-1})				
Rest	1.05 ± 0.31	1.11 ± 0.28	1.17 ± 0.30	1.11 ± 0.38†
0.67m s^{-1}	1.05 ± 0.19	0.84 ± 0.10*	1.25 ± 0.34	1.06 ± 0.23†
0.83 m s^{-1}	1.16 ± 0.22	0.87 ± 0.13*	1.37 ± 0.28	1.06 ± 0.26†
1.00 m s^{-1}	1.32 ± 0.27	0.91 ± 0.10*	1.30 ± 0.31	1.00 ± 0.27†
1.17 m s^{-1}	1.41 ± 0.33	0.92 ± 0.15*	1.39 ± 0.28	1.03 ± 0.17†
1.33 m s^{-1}	1.43 ± 0.31	0.96 ± 0.19*	1.51 ± 0.30	1.05 ± 0.14†
1.50 m s^{-1}	1.54 ± 0.32	1.00 ± 0.19*	1.47 ± 0.30	1.15 ± 0.18†
1.67 m s^{-1}	1.52 ± 0.25	1.18 ± 0.21*	1.67 ± 0.33	1.32 ± 0.19†

Results of a two-way ANOVA

	Normoxia	Hypoxia
Bf	Sex: F (1,18) = 4.19, *P* = 0.056	Sex: F (1,18) = 3.46, *P* = 0.079
	Speed: F (7,126) = 87.59, *P* < 0.001	Speed: F (7,126) = 132.47, *P* < 0.001
	Interaction: F (7,126) = 6.60, *P* < 0.001	Interaction: F (7,126) = 4.14, *P* < 0.002
TV	Sex: F (1,18) = 19.44, *P* < 0.001	Sex: F (1,18) = 11.84, *P* = 0.003
	Speed: F (7,126) = 11.71, *P* < 0.001	Speed: F (7,126) = 6.25, *P* < 0.001
	Interaction: F (7,126) = 6.04, *P* < 0.001	Interaction: F (7,126) = 1.51, *P* = 0.168

Values are mean ± SD. A two way repeated measures analysis of variance (ANOVA) was performed for a comparison between men and women within normoxia or hypoxia, respectively. * *P* < 0.05 between men and women in normoxia. † *P* < 0.05 between men and women in hypoxia.
